# Supplementary material for: Are carbon emissions trading and green financial instruments synergistic? -Comprehensive quantitative research based on content analysis
Source: PLoS One. 2024 Mar 7;19(3):e0298601. doi: 10.1371/journal.pone.0298601 (PMC10919601; doi:10.1371/journal.pone.0298601)
Supplement: S1 Data — (ZIP) [file pone.0298601.s001.zip › Data/地级市Z_F工作报告.zip!/╝¬┴╓/╦─╞╜╩╨/└┤╫╘╣1⁄2╓┌║┼í╛┬φ┐╦╩2╛▌═°í┐_2018.docx]

各位代表:

　　现在，我代表四平市人民政府，向大会报告工作，请予审议，并请市政协委员提出意见。

　　一、过去一年工作回顾

　　2017年是新一届政府的开局之年，也是实施“十三五”规划的重要一年。一年来，在省委、省政府的正确领导下，按照市委的部署，在市人大及其常委会和市政协的监督支持下，克难攻坚、砥砺奋进，“美丽四平、幸福家园”建设日新月异。预计到2017年末，地区生产总值实现1276亿元，增长5%；市本级全口径财政收入实现63.6亿元，增长6.1%；固定资产投资完成890亿元，增长6.5%，其中政府性投资增长20.4%；社会消费品零售总额完成650亿元，增长8%。

　　——经济运行稳中向好。项目支撑强劲有力。建立项目领办代办、投资项目并联审批和容缺承诺机制，实行春夏秋三季集中开工，全年建设5000万元以上项目186个，其中亿元以上项目133个。军民合用机场、地下综合管廊、君汇石油仓储物流、大清鹿苑产业园等一批重大基础设施和产业项目加快推进。经贸招商收获实效。赴京沪粤等地开展经贸合作交流，组织参加东博会等大型招商活动，积极推进换热器主题公园、专用车产业园等产业集群项目，成功举办换热器、专用车、农机及“百名工商企业家四平行”等高端峰会，谋划储备重点项目200个，纳入省招商引资调度系统3000万元以上项目到位资金265亿元，利用外资3.2亿美元。金融血脉更加通畅。工业、农业、服务业、城建等产业基金筹建工作扎实推进，中泰证券等5家金融机构入驻四平，金融街主体工程封顶，全年实现融资68.2亿元。创新源泉充分涌流。第二届中国·四平科技成果展洽会再结硕果，7个科技成果转移转化中心落户四平，“院士专家四平行”“双创”周等活动深入开展，新建院士工作站2个，新增省级科技“小巨人”企业5户、省质量奖企业2户，开展重大产学研合作60余项，中国农大、中科院长春应化所等创新平台快速推进，全市首次投产新产品122种。

　　——转型升级步伐加快。着力优化产业结构，三次产业比例达到27.2:36.7:36.1。供给侧结构性改革成效明显。“三去一降一补”重点任务有效落实，彻底拆除“地条钢”企业违规生产设备，化解房地产库存10.7万平方米，发行置换债券16.7亿元，降低企业税费5000余万元，企业生产经营成本攀升势头得到有效遏制。现代农业加速推进。粮食产量达到93.3亿斤，喜获“十四连丰”，“四平玉米”地理标志证明商标在国家正式核准注册，叶赫“卧龙白蘑”获国家地理标志产品保护。调减籽粒玉米107.5万亩，新增棚室生产面积2.3万亩，创建畜禽养殖标准化国家级示范园区3个，梨树高家村棚室园区发展模式全省推广。三产融合持续深化，全市农产品加工业总值突破500亿元。农民合作社、家庭农场分别发展到8304个、2715个，土地适度规模经营率达到56%。建设高标准农田37.7万亩，基本农田划定工作全面结束。建设省级农机全程机械化示范区12个，耕种收综合机械化水平超过85%；“黑土地论坛”效应持续释放，保护性耕作技术进一步推广，秸秆综合利用率达到70%左右；全省推进现代农业建设“三大体系”现场会在我市召开，四平模式为全省提供示范。工业动能转换全面提速。扶持“六个一百”重点企业快速发展。双辽离子膜烧碱装置整体搬迁等29个亿元以上产业项目开工建设。装备制造业持续做大做强，高技术产业快速增长。现代钢铁、金垦玻璃等15户“双停”企业启动生产。82户重点工业企业历史遗留问题得到解决。现代农业、换热器等“十大千亿级产业集群”朝目标扎实迈进。全年实施技改161项，技改投资增长10%。工业用电量增长9%。新增入规企业80户，规上工业增加值增长5.2%，增速位居全省第二位，四平工业脊梁正在挺起。商旅服务繁荣活跃。四平恒大综合体、万嘉购物广场等商贸项目进展顺利，智慧物流小镇等现代物流项目加快建设，伊通火山温泉度假区、双辽白沙温泉欢乐谷等旅游项目扎实推进。叶赫满族民俗旅游节、冰雪节、玉米节、龙舟赛等特色活动蓬勃开展，旅游总收入突破120亿元，伊通满族自治县被列为国家全域旅游示范区创建单位。我市国家级电子商务示范基地通过验收，电商企业突破200家，年交易额近60亿元。全年服务业增加值增长6.4%，拉动经济增长作用凸显。

　　——改革开放进一步提速。“放管服”改革持续深化。稳步推进简政放权，市本级取消行政审批事项16项，调整行政职权130项。加快商事制度改革，实施“多证合一”，新登记各类市场主体2.7万户，市场主体总数比2016年末增长10.5%，民营经济主营业务收入实现3000亿元。完成城市管理执法体制改革。整合建立市公共资源交易平台。推动“双随机、一公开”全覆盖，协同监管和联合惩戒机制基本形成。重点领域改革取得实效。城乡医保“三保整合”稳步推进，城市公立医院综合改革顺利实施，村医员额制管理创新开展。公务用车制度改革全面完成。供销合作社综合改革通过验收。试点县国有林场改革成效显著。农垦改革破题起步。乡镇站所管理体制改革率先发力。农村金融改革试点工作不断深化。对外开放步伐坚定有力。积极开拓国际市场，全年外贸出口增长11.3%。“航空飞镖”国际军事比赛保障工作圆满完成，四平知名度显著提升。分别与石家庄市、营口市及俄罗斯阿尔樵姆市缔结友好城市。与浙江金华开展全方位对接，签署对口合作框架协议。开发区建设全面提速，“十大增长极”成为经济增长重要引擎。

　　——城乡环境明显改善。生态环保工作取得重大进展。办理环保案件36批次、468件，打了一场漂亮的环境保护治理攻坚战。市区淘汰燃煤小锅炉166台，三年淘汰任务全部完成。淘汰黄标车6500辆。全年空气优良天数达到276天。“河长制”全面落实，“清河行动”成效初显，转山湖应急引水工程建成完工，南北河治理等工程稳步推进，架树台湖国家湿地公园成功获批。水源地保护力度持续加大，拆除水源地保护区违法建筑32处，新建保护区围栏2.1万米，彻底解决水源地保护历史遗留问题。市污水处理厂二期工程、郭家店镇污水处理厂、叶赫镇污水处理厂等项目基本完工，市区污水管网覆盖率和污水处理率分别达到85%、98%，出境河流水质有所改善。“户分类、村收集、镇转运、县处理”垃圾处理模式得到推广，建设垃圾中转站24个，市区永久垃圾分选厂建设全面启动，四平城区两处陈腐垃圾历史遗留问题得到有效破解。大力推进“四绿工程”，全年造林82.7万亩。一年来，我们锲而不舍抓环保，大刀阔斧除积弊，持之以恒强生态，美丽四平建设迈出新步伐。城乡基础设施建设成绩斐然。国道绥沈公路服先桥至金宝屯段交工通车，新建农村公路267公里、市政道路18条，长平高速公路出口改造完成。东丰路立交桥开工建设，紫气大路立交桥引桥部分进入尾声。改造提升主要街路风貌13条，建设海绵城市小区70个。新建续建地下综合管廊20.4公里，吉林省地下综合管廊产业联盟落户四平。市体育馆开馆运营，市图书馆即将竣工，一江山岛公园、西湖湿地公园对外开放。深入开展城乡环境综合整治专项行动，我市再次被确认为国家卫生城市。美丽乡村建设扎实开展。评选美丽庭院1万个、干净人家3万户，改造完成农家厕所16250户，全新打造3条精品线路，18个村晋级省级美丽乡村，铁东区叶赫满族镇入选第二批全国特色小镇，农村环境明显改善。

　　——民生福祉日益增进。35件民生实事取得实效，一批群众关注的热点难点问题得到解决。脱贫攻坚取得阶段性成果。危房改造完成6071户，60个贫困村安全饮水问题得到根本解决，16153名贫困人口实现脱贫，全市贫困发生率由5.1%降低至1.5%。就业创业和社会保障工作扎实推进。城镇新增就业4万人，农村劳动力转移就业42.5万人，城镇登记失业率控制在3.6%以内。发放创业担保贷款9600万元、扶贫贷款2850万元。社会保障提标扩面，农村低保标准首次超过省定扶贫标准。免费为34157名贫困人口办理城乡居民养老保险，发放社保补贴6338万元。养老服务持续规范，关停非法民办养老机构59家。安居工程稳步推进，棚改完成5026套，建成廉租住房444套。“无籍房”整治取得实效，291栋22792户住房进入不动产登记程序。社会事业全面进步。全市义务教育基本均衡发展通过国家验收，城市大学区管理深入实施，中小学生示范性综合实践基地建成并投入使用。强力推进“健康城”建设，深入实施“天使行动”计划，居民医疗保障水平得到有效提升。文化事业加快发展，相继举办首届“白山黑水·美丽四平”全国中国画作品展、第二届中国·四平满族文化论坛、二人转艺术节等活动，“千人同唱二人转”“万人旗袍秀展演”入选吉尼斯世界纪录。全市共有12部（件）艺术作品参加国家级（国际）展演展览。全民健身活动深入开展，全国百城自行车赛、全国太极拳公开赛成功举办。我市荣获“全国未成年人思想道德建设先进城市”称号。市公安局等12家单位被评为国家级文明单位。社会大局和谐稳定。治安形势持续好转，刑事案件发案率下降36%，侵财案件破案率位居全国前列，人民群众安全感明显提升。铁西区社区矫正工作先进经验在全国推广。安全生产领域改革稳步推进，安全生产监管责任体系逐步健全，安全生产形势保持稳定。食品药品安全四级监管网络不断完善。信访秩序持续规范，圆满完成党的十九大、全国“两会”、“一带一路”峰会和省十一次党代会等重大会事维稳任务，信访积案化解率达到85%。依法加强和改进民族宗教事务管理，民族团结、宗教和睦局面不断巩固。同时，妇女儿童、广播电视、知识产权、外事侨务、港澳台、人民防空、双拥共建、地震监测、气象、通信、老龄、地方志、残疾人等各项事业得到全面发展。

　　——政府自身建设不断加强。深入学习贯彻党的十九大精神，扎实推进政府系统“两学一做”学习教育常态化制度化，政治意识、大局意识、核心意识、看齐意识不断增强。自觉接受人大、政协和社会各界监督，认真执行人大及其常委会决议决定，主动向政协通报重大事项，127件人大代表建议、190件政协委员提案全部办复，满意率100%。加强依法行政，严把重大决策、规范性文件审查关，开展全市年度行政执法检查，法律顾问团“外脑”作用日益彰显。政风建设扎实有效，巩固和扩大巡视“回头看”整改成效，持续整治“四风”隐形变异问题。严肃治理“吃空饷”，清理长期旷工、事假、病假人员405人。监察工作得到加强，审计全覆盖稳步实施，法治政府、廉洁政府和服务型政府建设取得新进展。

　　各位代表！过去的一年，我们在经济下行压力加大、困难风险挑战增多的情况下，取得这些成绩实属不易。这是省委、省政府和市委正确领导的结果，是市人大、市政协监督支持的结果，是全市上下团结奋斗、苦干实干的结果。在此，我代表市政府，向广大干部群众，向人大代表、政协委员、各民主党派、工商联、无党派人士、人民团体和离退休老同志，向全市政法干警、驻平人民解放军、武警官兵，向驻平中省直单位，向参与和支持四平发展的各界朋友，表示衷心的感谢和崇高的敬意！

　　在肯定成绩的同时，我们也清醒地看到，我市经济社会发展还存在不少困难和问题：经济总量不大，产业结构不优，工业稳增长动力不足，创新能力不强，县域经济存在诸多短板。基础设施建设滞后，资源环境承载力有限，各种安全生产潜在风险依然存在。民生事业欠账较多，脱贫攻坚还不够精准。财政收支矛盾突出，政府偿债压力和债务风险不断增大。个别部门门好进、脸好看，但事难办，服务群众水平有待提升。部分干部不担当不作为，表态多调门高、行动少落实差，追责问责力度不够，一些决策部署落不了地。我们将高度重视这些问题，进一步强化责任意识，以担当进取的作风、革故鼎新的勇气，开创政府工作新局面。

　　二、今年主要目标及重点工作

　　2018年是全面贯彻落实党的十九大精神的开局之年，是决胜全面建成小康社会的关键之年，也是国家改革开放四十周年和四平解放七十周年，做好今年工作意义重大。政府工作总体要求是：以习近平新时代中国特色社会主义思想为引领，认真贯彻党的十九大精神、省委十一届二次全会精神，牢固树立和贯彻落实新发展理念，按照市委七届三次全会确定的打基础、促崛起、谋振兴“三步走”战略安排，坚持质量第一、效益优先、稳中求进，纵深推动“五路并进”“五市联动”“五城联创”“五个率先”，以供给侧结构性改革为主线，突出创新转型，加快动能转换，补齐发展短板，统筹推进稳增长、促改革、调结构、惠民生、防风险各项工作，加快建设新时代“美丽四平、幸福家园”。

　　全市经济社会发展的主要预期目标是：地区生产总值增长6.5%左右；地方级财政收入增长6.5%左右；工业增加值增长7%左右；社会消费品零售总额增长8.5%左右；单位GDP能耗下降3.3%；城乡居民收入增长与经济增长基本同步。

　　为实现上述目标，重点抓好以下九个方面工作：

　　（一）深入学习贯彻党的十九大精神。把学习贯彻党的十九大精神作为首要政治任务，坚持学原著、读原文、悟原理，准确把握习近平新时代中国特色社会主义思想实质，在学懂弄通做实上下功夫。深入推进党的十九大精神进企业、进农村、进机关、进校园、进社区、进军营、进网络，建立并充分发挥“新时代传习所”作用，开办政府网站学习专栏，发挥主流新闻媒体和“两微一端”等新媒体平台作用，持续掀起学习宣传贯彻十九大精神热潮。政府系统的领导干部要切实发挥关键少数作用，带头学、带头讲、带头做，锻造过硬作风，练就高强本领，坚持学以致用、知行合一，以学促干、以干践学，一心一意谋发展，心无旁骛惠民生，以实干推动振兴发展，靠奋斗实现美好生活，切实让党的十九大精神在四平落地生根。

　　（二）全力保持经济稳定增长。深入贯彻新发展理念，完善提升“八大支撑体系”，把握好工作节奏和力度，持续推动经济发展稳中求进、稳中向好。强力推进项目攻坚。全年计划建设5000万元以上项目150个,其中亿元以上项目100个。谋划储备新能源有轨电车等一批高质高效型项目，持续开展春夏秋三季项目集中开工，突出抓好天成玉米深加工异地搬迁扩能和新天龙玉米深加工扩能改造等重大项目。加快推进化工、医药、纺织、航空、军民融合、“双创”等园区建设，进一步提升项目承载能力和园区孵化能力。更加注重现有企业发展。始终把企业作为稳定四平发展的基石，鼓励支持企业释放产能、提升质量、开拓市场，做强做优做大实体经济，唱响工业强市主旋律。鼓励“六个一百”重点企业加快发展，支持烟厂、金士百、君乐宝、纳爱斯、立白等企业调优结构、增产增效，培育100户成长型企业升级入规，为工业发展持续注入新鲜血液。实施启动“双停”行动，通过吸引增量、出售产权股权、实施技改创新等措施，积极盘活40户“双停”企业，把资产闲置存量转化为经济增量。解决历史遗留问题，提升资本运作水平，引导企业通过债转股、引进上市公司参与股权重组，推动实体经济与金融资本合作、民营企业与高新技术融合。民营经济主营业务收入增长7%。务实精准招商引资。利用资源优势引资引智，依托现有企业以商招商，更加突出精准度、时效性和成功率。瞄准引进央企、大型民企和国内外500强企业，主动承接发达地区资本外溢和产业转移。利用中国国际服务贸易交易会、广交会等平台，助推本地企业拓展“两个市场”。举办好跨境电子商务峰会、进口商品展销会、全国六省市老字号企业展销会等系列活动。提升要素保障能力。落实领导包产业、包项目、包园区责任制，全力破解瓶颈制约。开展多元化融资，吸引各类金融机构入驻，着力培育普惠金融，重点解决企业贷款难问题，推动我市企业上市挂牌进程。提高土地供应水平，盘活土地存量，加大收储力度，依法依规收回闲置建设用地。增加人才吸引力，大力培养创新型人才，发挥硕博人才作用，弘扬“工匠”精神，加快打造“科技四平”“创新四平”。

　　（三）加快产业转型升级步伐。转变发展方式，优化经济结构，大力培育新动能，持续提升经济发展质量和效率，加快构建四平现代化经济体系。推进供给侧结构性改革。坚决打破拖累转型升级的“坛坛罐罐”，抓实“三去一降一补”，提高供给质量，增强供给能力。扎实推进金融服务实体经济、防控金融风险、深化金融改革三项主要任务。抓住省支持发展四平金融功能区机遇，加快金融集聚区、金融街建设。切实加强政府债务管理，严格限定举债程序和资金用途。做好金融风险重点环节监管，严厉打击违法违规金融活动，防范化解资金链、担保链风险。全面落实取消涉企行政事业性收费等政策措施，大力降低企业成本。着力补齐发展短板，继续实施推进一批教育、医疗、交通等公共服务类项目。坚持创新驱动发展战略。瞄准中国制造2025，加快培育智能制造、光电产业、干细胞应用、工业物联网等新业态。发挥6家院士工作站和12个科技成果转移转化中心作用，深入开展“院士专家四平行”等活动，引导资金、技术、项目、人才等创新要素向企业集聚，为企业突破关键技术、创建高端品牌提供智力支持和技术支撑。强化产学研协同创新，推动吉林省换热器产业创新中心建设，鼓励企业加大研发投入，培育一批具有创新能力的“排头兵”企业。新增高科技“小巨人”企业20户以上。优化提升传统产业。持续举办高端峰会，推进换热器、专用车、农机等千亿级产业集群发展，加快先进装备制造、农产品加工等主导产业升级改造进程，巩固提升“三四三”特色产业体系。深入落实工业转型升级行动计划，加快新一轮技术改造，推进头孢抗生素万能成盐剂等一批创新技改型项目投产达效。全年技改投资增长10%。大力发展现代服务业。加快服务业业态创新、模式创新和产品创新，推动现代物流、电子商务、互联网金融等产业发展。优先发展生产性服务业，推进智慧物流小镇、浙江新农等项目建设。着力发展生活性服务业，加快欧亚商业综合体、万嘉购物广场、五洲国际商贸城等项目建设。支持发展共享经济、体验服务等新兴服务业态。实施旅游产业差异化发展，以“中国·四平国际旅游节”为引领，做足红色、满族、冰雪、生态等特色文章，全力推动二龙湖国际旅游度假区、伊通火山温泉度假区、双辽一马树森林公园等景区提档升级，谋划建设叶赫故宫学院、中华美食城、啤酒广场等项目，培育打造叶赫满族“冰嬉节”等旅游品牌，持续开展英城嘉年华等特色活动，打造一批具有四平特色的旅游精品项目。

　　（四）扎实推动乡村振兴。按照产业兴旺、生态宜居、乡风文明、治理有效、生活富裕总要求，科学制定四平乡村振兴规划，纵深推进农业立市，加快构建现代农业“三大体系”，全面提升土地产出率、资源利用率、劳动生产率，推动农业升级、农村进步、农民发展，推进农业农村现代化建设。做强农业产业。因地制宜发展优质水稻、专用大豆、酒用高粱等高效作物，打造梨树100万亩玉米、双辽20万亩杂粮杂豆国家绿色食品原料标准化生产基地。大力发展棚膜经济、庭院经济等特色产业，新增棚膜面积4万亩，新建扩建棚室园区20个。健全良种繁育、规模养殖、屠宰加工体系，标准化规模养殖场（小区）数量达到350个，畜禽规模化养殖比重达到75%。加快建设优质农产品展销中心和农产品网上商城，开通“惠农通”手机APP客户端，推进农产品追溯体系建设，探索智慧农业发展模式。推动一二三产融合发展，助推天成玉米等企业转型升级，谋划好丛泉湖休闲农业等一批特色项目，提升完善采摘节等乡村游活动，促进玉米产业化、秸秆产业化、农业品牌化。农产品加工业总值实现550亿元。做优农业生产。巩固和提升粮食产能，实现藏粮于地、藏粮于技，全年粮食产量稳定在90亿斤阶段性水平。普及应用测土配方施肥、免耕播种等新技术，总结推广“稻蟹（鱼）共生”、多种轮作等新种植模式，着力推动高标准农田建设。提高全程机械化生产覆盖率，建成50个高标准全程机械化示范区。做活农业经营。围绕农民增收这个核心，进一步鼓励发展流转、托管、入股等多种形式的适度规模经营，加快培育壮大百户新型经营主体，土地适度规模经营率达到60%以上。探索“资源变资产、资产变股金、农民变股东”发展路径，加大村集体项目以奖代补、贷款贴息、风险担保等支持力度，落实税费、土地、金融支持政策，保障农民财产权益，持续壮大农村集体经济。提升新农村品质。巩固农村环境整治成果，加快推进农村“五改”，探索资金整合投入方式，建立村容保洁长效机制，扎实推进安全饮水、电网升级、光纤入户、厕所改造及村镇医疗、文化、体育设施等工程建设，新建农村公路150公里，大力提升农村基本公共服务水平。深化乡风文明建设，新增美丽乡村60个、精品线路35条。

　　（五）坚定不移深化改革扩大开放。增强改革定力，保持改革韧劲，推动形成改革开放新格局。加大“放管服”改革力度。坚持真改、真放、真活，以更大决心推进简政放权，增强市级决策统筹和市辖区执行落实能力，今年在进一步调整和完善行政审批事项、权力清单、责任清单基础上，还将向两区、开发区下放一批行政职权，实现重心下移、财力下沉、权责匹配、效能提升。进一步优化发展环境，不断完善并联审批、现场审批、领办代办、容缺承诺等模式，努力在加强最优制度供给、营造最佳营商环境上更进一步。加快建设政务服务“一张网”，探索推进“零见面网上办”“最多跑一次”机制，最大限度实现利企便民。深化商事制度改革，推进“证照分离”，打造宽松便捷市场准入环境，深化“双随机、一公开”监管方式，提高监管有效性。推动“诚信四平”建设，加快构建亲清新型政商关系，激发企业家创新发展的热情，营造企业竞相发展的良好环境，真正使四平成为投资创业的热土。统筹实施重点领域改革。稳妥推进农垦改革，组建辽河农垦集团公司，实现垦区集团化、农场企业化、社会管理属地化改革目标。巩固公立医院综合改革、基层医疗卫生综合改革成果，持续推动城乡医保管理体制改革，全面抓好医改任务落实。推进事业单位分类改革，优化机构编制资源配置。深化水务一体化体制改革，提升水资源综合利用水平。抓好财税体制改革，加快建立现代财政运行机制体系。实施投融资体制改革，推动政府投融资平台向市场化转型。继续推进乡镇站所管理体制改革，完成农村集体产权制度改革试点任务。深化农村金融改革，设立现代农业贷款风险基金。提高对外开放水平。鼓励优势重点企业到“一带一路”沿线国家洽谈合作、建园设厂，深化与国际友城之间的合作，进一步优化我市外贸结构。认真落实哈长城市群规划和长平经济合作区发展战略，积极推进“四辽铁通”次区域合作。实施四平金华对口合作三年计划，谋划推进北方物流基地、浙商“区中园”等项目，发展“飞地”经济，以更大的开放推动更快的发展。

　　（六）全面打赢生态环保战役。践行“绿水青山就是金山银山”理念，坚持对大气、水、土壤污染零容忍，巩固提升环保问题整改成果，使主要污染物排放总量大幅减少，生态环境质量总体改善。打好蓝天保卫战。持续加大对钢铁、水泥、化工、火电等重点行业整治力度，加强秸秆焚烧监管，开展餐饮服务业油烟治理，抓好建筑工地扬尘管控，做好重污染天气应急处置，确保PM10、PM2.5、空气优良天数、重污染天数等四项指标越来越好。打好水体攻坚战。坚持科学治水、节约用水、依法管水，统筹做好四平城市水系治理及水资源综合利用。完善做实“河长制”“湖长制”，实现河湖巡查常态化，消除河湖流域内非法畜禽养殖、偷排乱排等行为。深入实施清河行动，着力推进河湖连通、东辽河和伊通河治理、黑臭水体整治等重点工程建设，实现水质明显改善，确保出境断面水质和二龙山水库水质达到国家考核要求，让每一条河流都成为美丽四平的风景线。打好土壤修复战。加强农业面源污染防治，实施农药和化肥使用量负增长行动，大力推行秸秆还田，抓好畜禽养殖废弃物处理和资源化利用，彻底清理陈腐垃圾，推动垃圾减量化、资源化、无害化，为子孙后代守好这片宝贵的黑土地。打好生态保护持久战。划定并严守生态保护红线，持续推进“四绿工程”，启动农村防护林修复工程，完成四平环城路绿化带建设，推进城区园林景观绿化改造提升，实现50万亩以上植树造林任务。大力实施山水林田湖草生态保护和修复工程，清收林地30万亩以上。强化生态敏感区治理保护，加快推进架树台湖国家湿地公园建设，加大半拉山国家级森林公园、山门中生代火山国家级自然保护区和下三台饮用水水源地保护力度，提升自然生态系统质量和稳定性。突出加强环境保护执法能力建设，严厉打击各类违规违法行为，始终保持严查、严处、严打的高压态势。

　　（七）着力推动城乡融合发展。围绕打造国家二类大城市，纵深推进“五城联创”，加强城乡建设管理，提高城乡品质，推动融合发展，着力建设具有新时代风貌的英雄城。加强城乡统筹规划。坚持规划引领发展，全面启动四平市城市总体规划修编，制定特色城镇总体规划和美丽村庄建设规划，统筹城乡空间有序发展、产业融合发展、设施均衡发展、社会和谐发展、生态联动发展，建设具有四平特色的城乡规划体系。提高城乡建管水平。加快推进“十大历史性工程”，完善城市功能，市图书馆、滑冰馆交付使用，市博物馆、大剧院主体完工。优化市区路网结构，紫气大路立交桥、东丰路立交桥实现竣工通车，康平街公铁立交桥、四梅线三座平改立下穿式立交桥项目年内开工建设。加强城市立体综合开发，新建地下综合管廊16公里，管线入廊45公里。推动内畅外联互通，加快推进军民合用机场项目，实现国道牙四公路（桑树台至四平段）、二龙湖至叶赫旅游公路、四平至杨木林公路主体工程通车，完成双嫩高速公路土地征拆工作，推动二环路、四平至伊通公路项目开工建设。建立市县乡村屯五级联动爱国卫生管理体系，巩固国家级卫生城市成果，强力开展城区环境集中整治行动，进一步规范小区物业管理，拆除各类违法建筑，继续实施楼体亮化提升和风貌改造工程，完成东山公园主体工程建设，实现南湖公园二期年内开园，打造靓丽宜居城市。推动城市近郊率先融合。坚持以工补农、以城带乡，全力做大做强城市发展中心区、城乡融合拓展区、现代农业建设区。释放四平主城区辐射带动作用，新开区、龙湖区、铁北生态新区发挥产业优势，强壮经济骨骼，连通城乡血脉。城东乡、平西乡完成撤乡建镇工作，叶赫满族镇、山门镇依托自身资源优势，建设宜居宜业宜游的新城区和特色小镇。清除阻碍要素下乡各种障碍，鼓励农民进城置业创业，推动农村人口城镇化。支持梨树县、伊通满族自治县、双辽市发挥优势、形成特色，加快补齐县域短板。

　　（八）更大力度保障和改善民生。坚持以人民为中心发展思想，推进民生保障与社会事业同步提升，不断满足人民日益增长的美好生活需要。精准推进脱贫攻坚。更加注重精准和质量，因村因户施策，创新产业扶贫，落实教育扶贫，强化健康扶贫，用活金融扶贫，注重扶贫与扶志、扶智相结合，激发贫困人口内生动力，完善更系统、更严格的考核评估、督查巡察、资金监管、驻村工作等管理制度，确保脱真贫、真脱贫，全年实现脱贫9262人。提高就业创业水平。实施更加积极的就业政策，着力构建创业促进体系，加快推进吉林师大双创大厦等项目建设，打造一批众创空间、大学生创业园、农民工返乡创业孵化基地，降低群众创业创新成本。统筹做好各类群体就业工作，落实好援企稳岗、社保补贴、税费减免等政策，全市城镇新增就业3.2万人，农村劳动力转移就业35万人次。织密社会保障网底。加快全民参保步伐，提高保障标准和服务水平，打造智慧医保，加大社会救助力度，推进城市脱困攻坚，建立稳定脱困常态化长效化机制，着力构建多层次社会保障体系。落实养老服务普惠计划，加快老龄事业和产业发展，新建10所老年公寓。抓好棚户区、保障房和农村危房改造等项目建设，让老百姓切实享受到惠民政策带来的实惠。统筹发展社会事业。以创建全国文明城市为统领，积极培育和践行社会主义核心价值观，广泛开展精神文明创建活动，大力弘扬“高尚、智慧、创新、卓越”的新时代四平精神，赋予英雄城新的时代内涵。积极支持文化产业发展，提供更优更多公共文化产品，举办好庆祝四平解放七十周年系列活动，创作一批具有四平特色的精品力作。推进教育充分协调发展，深化中考高考改革，集中开展师德师风专项整治行动，着力解决中小学生课外负担重等突出问题，打造四平特色职业教育品牌。广泛开展全民健身活动，加快体育产业发展。纵深实施“天使计划”，积极推进中医院、儿童医院和妇产医院建设，结核病防治所、疾控中心、食药检所建成使用，建立医疗机构运行新机制，提升中医药水平，继续解决好“看病难、看病贵”问题。新建4G以上基站320座，实现城乡宽带全覆盖。同时，统筹推进妇女儿童、人民防空、民族宗教、双拥共建、残疾人等各项事业发展。

　　在做好各项民生工作同时，重点推进十项民生工程：一是建设高标准幼儿园16所，努力满足幼教发展需求；二是完成市区棚户区改造4300套，继续改善市民住房条件；三是解决市区历史遗留7万户“无籍房”问题，切实维护群众合法权益；四是完成市区海绵城市80个小区改造任务，不断优化市民居住环境；五是改造市区15个自备水源小区供水管网，确保居民饮水安全；六是打通市区街路18条，方便百姓出行；七是新建改建城乡公厕200座，全力推进“厕所革命”；八是建成市区永久性垃圾分选厂，保障生活垃圾得到及时处理；九是在市区重点区域安装火灾报警器3000个，提高火灾预警能力；十是规划建设四平公益生态公墓，提升殡葬服务水平。

　　（九）切实维护社会和谐稳定。完善社会治理体制，提升社会治理水平，深入实施“七五”普法规划，加速依法治市建设进程，进一步打造共建共治共享的社会治理新格局。加强社会治安综合治理。创新完善立体化、信息化社会治安防控体系，建立公共法律服务平台，健全公共安全风险隐患排查治理和应急处置机制。深入开展打黑除恶专项行动，依法惩治盗窃、抢劫、电信网络诈骗等多发性犯罪，实施打击毒品违法犯罪、制贩枪支犯罪和寄递物流安全管理专项整治，始终保持严打高压态势。推进信访法治化建设。提高信访工作专业化、法治化、规范化水平，围绕控总量、减存量、遏增量，集中开展重点领域信访攻坚，完善群众权益保障机制、群众利益诉求表达收集反馈机制和依法分类处置信访诉求工作机制，增强处理复杂问题、做好群众工作能力，实现信访秩序根本好转。狠抓公共安全管理。树牢安全发展理念，严格落实“党政同责、一岗双责、齐抓共管、失职追责”工作要求，压实企业主体责任，深化市县乡村四级监管网络建设，加强对重点场所、重点行业领域、重点企业的隐患排查整治，坚决遏制重特大安全事故发生。健全食品药品质量安全治理保障体系，实行最严格的食品药品安全监管制度，让人民群众吃得放心、喝得安全。

　　三、加强政府自身建设

　　打铁必须自身硬。实现全年目标任务，必须进一步增强改革创新、科学发展、狠抓落实等本领，加强学习和实践，提高职业素质，培养专业能力，全力打造担当干事、纾难解困、务实高效的执行团队，在新时代展现新气象、实现新作为。

　　加强依法行政。政府系统的所有行政行为都要于法有据。全面贯彻落实《法治政府建设实施纲要》，强化法治思维，带头学法尊法守法用法，始终牢记法律红线不可逾越、法律底线不可触碰。严守规矩程序，自觉接受人大依法监督、政协民主监督及各方面的监督，持续规范行政许可、行政处罚、行政强制、行政征收、行政检查等行政执法行为。提升执法效果，更加突出问题导向和目标导向，对于该管的事情，坚决管住管好，对于各类违法行为，决不姑息放纵，真正做到法无授权不可为、法定职责必须为。

　　提升服务效能。服务能力和服务效率是建设服务型政府的关键所在。强化服务理念，把群众的事放在心上，把群众的小事当成政府的大事，围绕“事要办成”这个关键，不仅门好进、脸好看，更要事好办、办好事、办成事，真心实意为群众生活排忧解难。提升服务质量，完善政务服务体系，优化政务服务流程，把更多精力放在事中事后监管上，对企业遇到的问题，多提供可行可办的建议意见，多研究破解障碍的措施办法，理直气壮为企业家创业发展撑腰打气。

　　打造过硬作风。作风建设永远在路上。增强创新意识，善于谋划，勇于变革，用工作的主动性、创造性打开新局面。增强责任意识，不拖不躲、不等不靠，对重大工程和重点工作要明晰责任，跟踪督查调度，不达目的不罢休。增强求实意识，少开会、开短会，大兴调查研究之风，掌握真实情况，找准短板弱项，解决实际问题。市政府党组成员要以身作则、以上率下，形成头雁效应。

　　建设廉洁政府。巩固深化党的群众路线教育实践活动成果，深入践行“三严三实”，扎实推进“两学一做”学习教育常态化制度化，根据中央精神认真开展“不忘初心、牢记使命”主题教育。坚决扛起从严治党政治责任，严守政治纪律和政治规矩，严格执行中央八项规定及《实施细则》，坚决防止纠正形式主义、官僚主义新表现，持续深化“两整治、两增强”专项行动，加大追责问责力度，持之以恒正风肃纪。以零容忍态度惩治腐败，健全监督体系，构筑起不敢腐、不能腐、不想腐的坚固防线。

　　各位代表！使命呼唤担当，实干成就未来。让我们紧密团结在以习近平同志为核心的党中央周围，在市委坚强领导下，在市人大、市政协监督支持下，坚定信心，锐意进取，埋头苦干，为加快建设新时代“美丽四平、幸福家园”而努力奋斗！
